# Supplementary material for: Curcumin and Ethanol Effects in Trembler-J Schwann Cell Culture
Source: Biomolecules. 2022 Mar 29;12(4):515. doi: 10.3390/biom12040515 (PMC9025918; doi:10.3390/biom12040515)
Supplement: Supplementary file 1 [file biomolecules-12-00515-s001.zip › biomolecules-1635132-supplementary.pdf]

biomolecules

MDPI

Curcumin and Ethanol Effects in Trembler-J Schwann Cell Culture

In this supplementary material, we present our immunolabeling controls and preliminary results about DMSO effects on endoneural fibroblasts wild-type (+/+) cultured, obtained for sciatic nerve. Also, we provide evidence found by our group regarding mitochondrial differences between Trembler-J (TrJ/+) mice, a murine model of CMT1, and +/+ mice. This data presented were performed from nerves of +/+ and TrJ/+ mice.

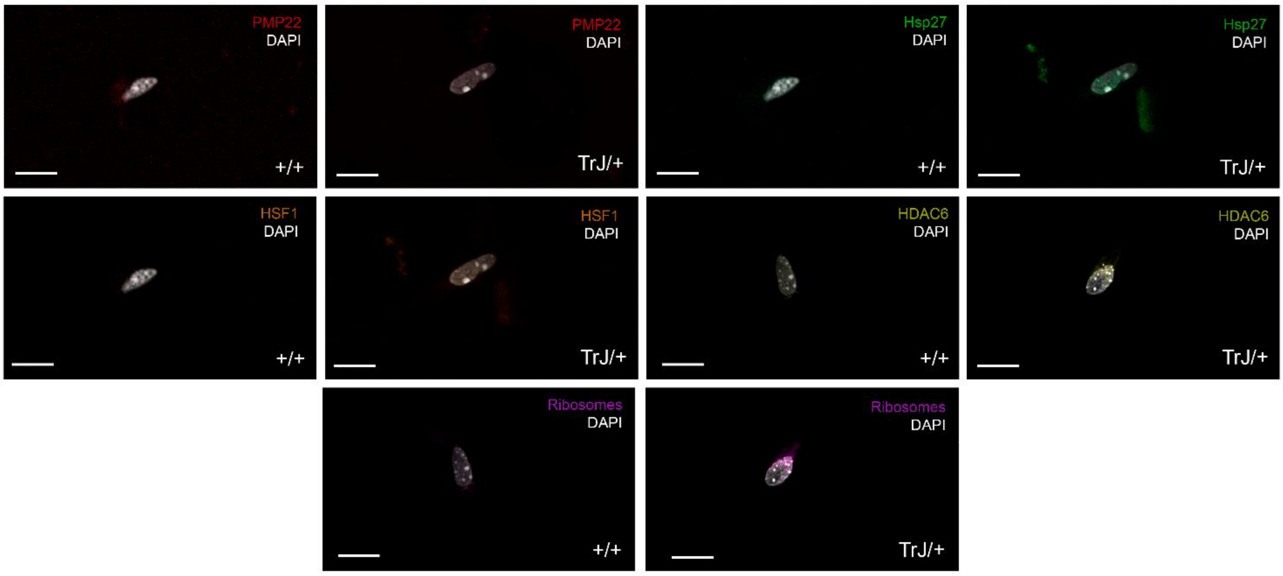

Figure S1. Immunolabeling controls. Negative controls for PMP22, Hsp27, HSF1, HDAC6, and ribosomes markers are shown for +/+ and Tr/J SCs culture. In all cases we obtained an unspecific labeling. Scale = 20 µm for all panels.

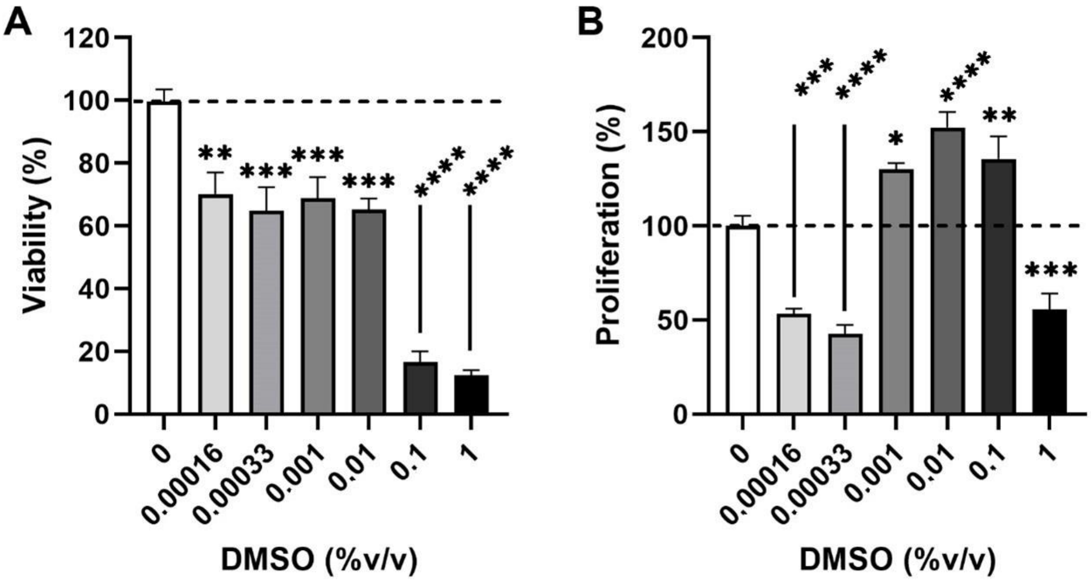

Figure S2. Effect of DMSO on the viability and proliferation of endoneural fibroblasts +/+ cultures. **(A)** Viability was assessed by MTT assay. The differences observed after five days or treatment translate into a decrease in cell viability ($F_{(6,56)} = 1{,}950$). Significant differences against the untreated control were observed with 0.00016% (p = 0.0011), 0.00033% (p = 0.0001), 0.001% (p = 0.0007), 0.01% (p = 0.0001), 0.1% (p < 0.0001) and 1% (p < 0.0001). **(B)** Proliferation was assessed by CyQUANT proliferation assay ((#cat. C7026, Invitrogen, Thermo Fisher Scientific, USA). After five days of treatment, all concentrations analyzed were differences respect to untreated control ($F_{(6,56)} = 3{,}987$): 0.00016% (p = 0.0001), 0.00033% (p < 0.0001), 0.001% (p = 0.0266), 0.01% (p < 0.0001), 0.1% (p = 0.0055) and 1% (p = 0.0003). Data were evaluated by a one-way ANOVA test. *p < 0.05, **p < 0.002, ***p < 0.0002, ****p < 0.0001.

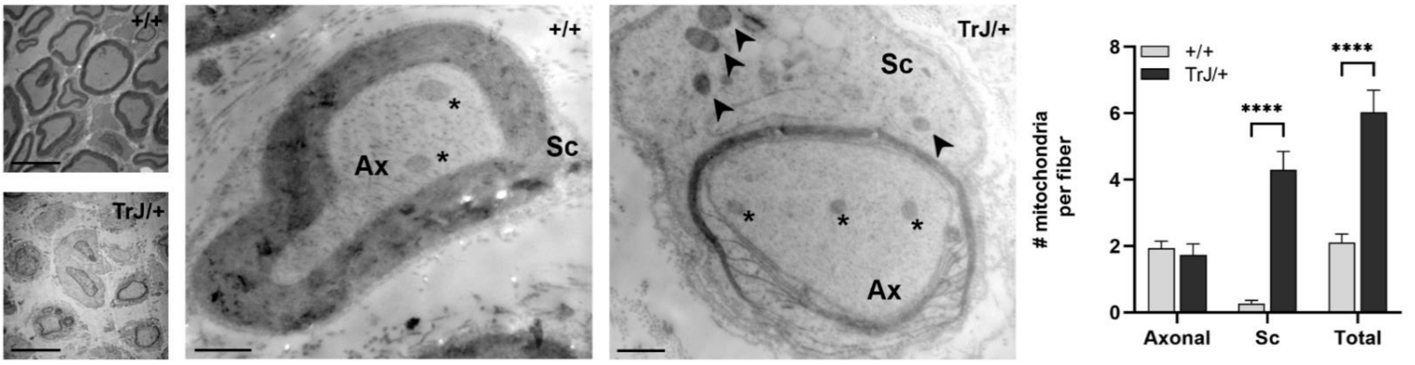

Figure S3. Mitochondrial distribution in fibers of TrJ/+ and +/+ mice. From electron microscopy images, the number of mitochondria per fiber was quantified, discriminating between the axonal domain (Ax) and the Schwan cell domain (Sc). The count of total mitochondria per fiber allowed us to determine a greater number of this organelle in TrJ/+ fibers. The discrimination of mitochondria according to their domain (arrowhead SC and * Ax) allowed us to understand the differences between TrJ/+ and +/+ is at the SC level since it is the only domain where we found significant differences (Total: U = 206.5, p < 0,0001; Sc: U= 120.5; p < 0.0001). Scale in small images = 3 μm. Scale in large images = 500 nm. **** p < 0.0001.

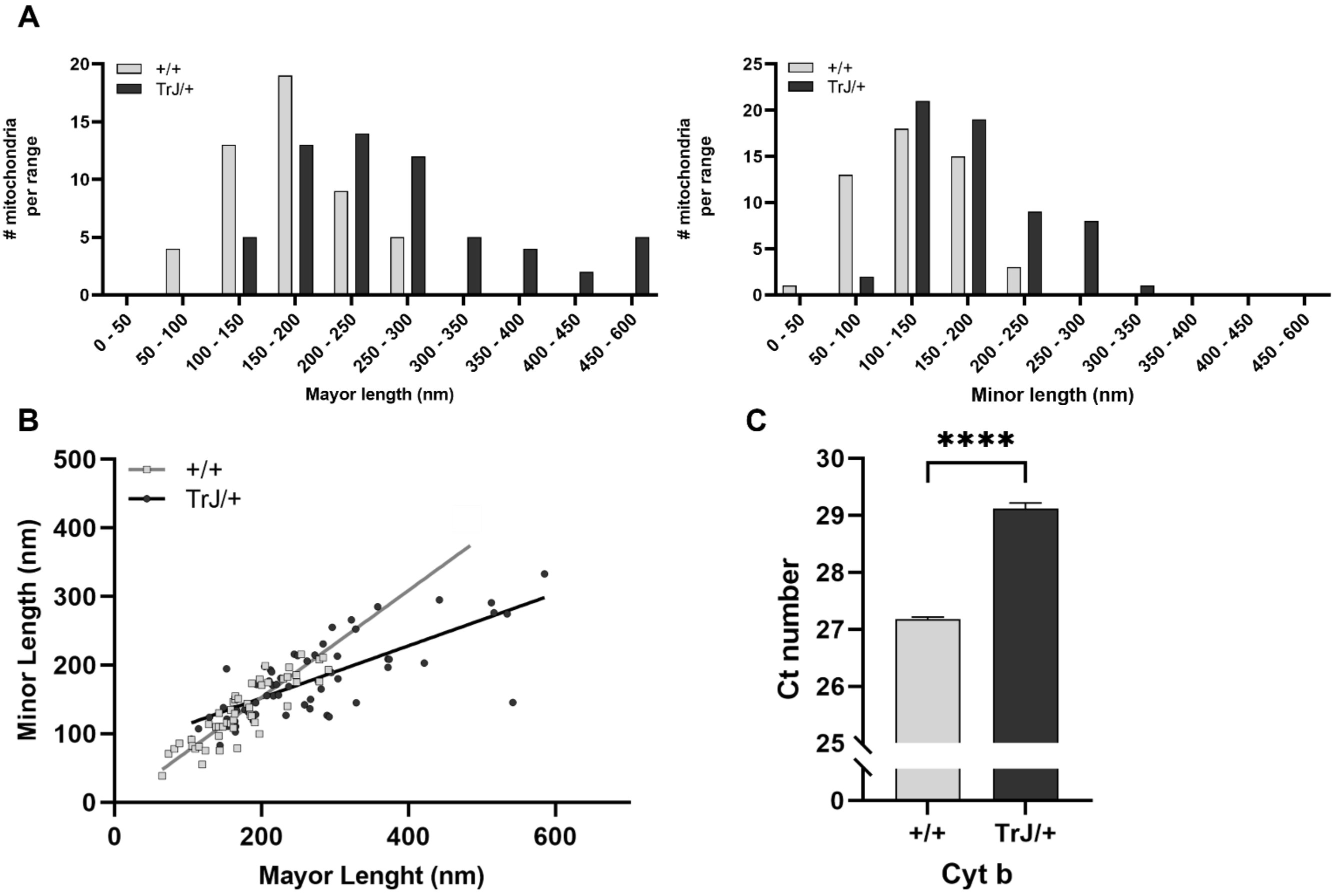

Figure S4. Morphology and differential gene expression in mitochondria of SCs from TrJ/+ and +/+ mice. Schwann cell mitochondria were analyzed to understand some of their morphology, cytochrome b transcriptional expression and differences between +/+ and TrJ/+ genotypes. (**A**) The distribution of mitochondrial lengths (major and minor) was evaluated for +/+ and for TrJ/+. Sc TrJ/+ mitochondria present higher values of major diameter, reaching values of 450-600 nm, compared to +/+, where the largest diameter reached by mitochondria is 250-300 nm. We also found differences in the modal value of the distribution; while for +/+ mitochondrial the mode is in the range of 150-200 nm, for TrJ/+ it is 200-250 nm. (**B**) Correlation was found between major and minor lengths of mitochondria in +/+ and TrJ/+ genotypes ($p < 0.0001$). Linear regression of the data was performed, where both genotypes fit a straight line, but with different equations. (**C**) Cytochrome b expression levels showed a significant increase in Ct number in TrJ/+, compared to +/+ (t = 18.53, df = 8, **** $p < 0.0001$).
